# Supplementary material for: Correlation of Nutritional Indices on Admission to the Coronary Intensive Care Unit with the Development of Delirium
Source: Nutrients. 2018 Nov 8;10(11):1712. doi: 10.3390/nu10111712 (PMC6267104; doi:10.3390/nu10111712)
Supplement: Supplementary file 1 [file nutrients-10-01712-s001.pdf]

Table S1. Associations between nutritional Indices and several risk factors.

	GNRI		PNI		CONUT	
	r	P	r	P	r	P
Age, years	0.86	<0.001	0.29	<0.001	0.25	<0.001
Male, n (%)	0.16	<0.001	0.14	0.0003	0.06	NS
Body mass index, kg/㎡	0.40	<0.001	0.18	<0.001	0.17	<0.001
Left ventricular ejection fraction, %	0.08	NS	0.10	0.019	0.12	0.005
Diabetes mellitus, n (%)	0.04	NS	0.06	NS	0.08	NS
Dyslipidemia, n (%)	0.15	0.0002	0.16	<0.001	0.09	0.03
Hypertension, n (%)	0.01	NS	0.01	NS	0.03	NS
Atrial fibrillation, n (%)	0.10	0.011	0.14	0.0004	0.16	0.001
Dementia, n (%)	0.11	0.007	0.11	0.005	0.12	0.004
Cerebral infarction, n (%)	0.00	NS	0.02	NS	0.03	NS
Malignancy, n (%)	0.18	<0.001	0.17	<0.001	0.21	<0.001
**Laboratory data**						
Albumin, g/dL	0.93	<0.001	0.87	<0.001	0.78	<0.001
Total cholesterol, mg/dL	0.27	<0.001	0.28	<0.001	0.55	<0.001
Triglycerides, mg/dL	0.15	0.0002	0.18	<0.001	0.23	<0.001
HDL-C, mg/dL	0.19	<0.001	0.18	<0.001	0.33	<0.001
LDL-C, mg/dL	0.21	<0.001	0.22	<0.001	0.47	<0.001
Creatinine, mg/dL	0.15	<0.001	0.19	<0.001	0.19	<0.001
HbA1c, %	0.05	NS	0.04	NS	0.07	<0.001
CRP, mg/dL	0.45	<0.001	0.46	<0.001	0.49	<0.001
NT-pro BNP, pg/mL	0.23	<0.001	0.21	<0.001	0.21	<0.001

The association between each nutritional index and risk factors were assessed using the single liner regression analysis. HDL-C, high-density lipoprotein cholesterol; LDL-C, low-density lipoprotein cholesterol; HbA1c, national glycohemoglobin standardization program calculation; CRP, C-reactive protein; NT-pro BNP, N-terminal pro brain natriuretic peptide.
